# TnaA, an SP-RING Protein, Interacts with Osa, a Subunit of the Chromatin Remodeling Complex BRAHMA and with the SUMOylation Pathway in *Drosophila melanogaster*

**DOI:** 10.1371/journal.pone.0062251

**Published:** 2013-04-19

**Authors:** Juan Monribot-Villanueva, R. Alejandro Juárez-Uribe, Zoraya Palomera-Sánchez, Lucía Gutiérrez-Aguiar, Mario Zurita, James A. Kennison, Martha Vázquez

**Affiliations:** 1 Departamento de Fisiología Molecular y Genética del Desarrollo, Instituto de Biotecnología-Universidad Nacional Autónoma de México, Cuernavaca, Morelos, México; 2 Program in Genomics of Differentiation, Eunice Kennedy Shriver National Institute of Child Health and Human Development, National Institutes of Health, Bethesda, Maryland, United States of America; St. Georges University of London, United Kingdom

## Abstract

Tonalli A (TnaA) is a *Drosophila melanogaster* protein with an XSPRING domain. The XSPRING domain harbors an SP-RING zinc-finger, which is characteristic of proteins with SUMO E3 ligase activity. TnaA is required for homeotic gene expression and is presumably involved in the SUMOylation pathway. Here we analyzed some aspects of the TnaA location in embryo and larval stages and its genetic and biochemical interaction with SUMOylation pathway proteins. We describe that there are at least two TnaA proteins (TnaA_130_ and TnaA_123_) differentially expressed throughout development. We show that TnaA is chromatin-associated at discrete sites on polytene salivary gland chromosomes of third instar larvae and that *tna* mutant individuals do not survive to adulthood, with most dying as third instar larvae or pupae. The *tna* mutants that ultimately die as third instar larvae have an extended life span of at least 4 to 15 days as other SUMOylation pathway mutants. We show that TnaA physically interacts with the SUMO E2 conjugating enzyme Ubc9, and with the BRM complex subunit Osa. Furthermore, we show that *tna* and *osa* interact genetically with SUMOylation pathway components and individuals carrying mutations for these genes show a phenotype that can be the consequence of misexpression of developmental-related genes.

## Introduction

SUMOylation is a post-translational protein modification that can change the location, stability, activity or the interactions of the protein targets involved in many cellular processes, including cell death, cell cycle, signal transduction, and gene expression [1]. SUMOylation is the addition of SUMO (Small Ubiquitin-related MOdifier) to lysine residues of the target protein in the consensus amino acid sequence ΨKxE (Ψ represents a hydrophobic amino acid) [2]. Hundreds of proteins are SUMOylated in *Drosophila* [3]. The SUMOylation pathway starts with processing of an immature SUMO protein by the Ulp/SENP family of proteases. Next, the activating enzyme E1 (an Aos1/Uba2 heterodimer) generates a mature SUMO-adenylate intermediary which then forms a thioesther bond between the catalytic cysteine of Uba2 and SUMO. SUMO is next transferred to the E2 conjugating enzyme (Ubc9), which transfers SUMO to the target proteins. The SUMO E3 ligases function by stimulating the activity of Ubc9 or by facilitating the formation of an Ubc9-substrate complex. Finally, proteins of the Ulp/SENP family proteases make this whole process reversible [4].

The *tna* gene was identified in a genetic screen designed to find *brahma* (*brm*)-interacting genes [5]. *brm* encodes the SNF2 type-ATPase of the BRM chromatin remodeling complexes [6], [7]. The *osa* gene encodes an exclusive subunit of one type of BRM complexes [6], [8], [9]. Besides interacting with *brm*, *tna* interacts even stronger with *osa*. All three genes (*brm*, *osa,* and *tna*) are required for proper expressions of the homeotic genes [5]. Homeotic genes determine the identity of body segments in *Drosophila* [10], [11].

The role of various components of the SUMOylation pathway have been studied in *Drosophila* development [12], [13]. *tna* is involved in homeotic gene expression but little is known about the proteins encoded by this locus. *tna* expresses a at least one putative isoform called TnaA [5]. This isoform has an XSPRING (eXtended SP-RING) domain that harbors a zinc finger of the SP-RING type {Siz/PIAS (Protein Inhibitors of Activated STAT [Signal Transducers and Activator of Transcription])–RING (Really Interesting New Gene)}. This zinc finger is present in one of the four major groups of proteins that have SUMO E3 ligase activity [1]. The only SP-RING finger proteins with putative SUMO E3 ligase activity that have been identified in the *Drosophila* proteome are Su(var)2–10 [14] and TnaA [5].

Here we show that TnaA physically interacts with both Ubc9 (the SUMO E2 conjugating enzyme) and with Osa (a putative *in vivo* target). We determined the dynamics of different TnaA species throughout development and showed that TnaA is an embryonic nuclear protein and is also present at discrete bands on polytene salivary gland chromosomes of third instar larvae. We also found that defects in *tna* cause larval lethality, abnormalities in the whole protein profile and an extension of the lifespan at this stage. Finally, we found genetic interactions between *tna* and *osa* and genes encoding the SUMOylation pathway components.

## Materials and Methods

### Ethics Statement

All animal handling was approved by the Instituto de Biotecnología Bioethics Comittee, Permit Number 226 (2009/12/04), which follows NOM-062 animal welfare mexican law. All efforts were made to minimize animal suffering. Animals were sacrificed by CO_2_ euthanasia.

### Protein Extraction and Analyses

Soluble protein extracts for the developmental Western were obtained from 1 g of Ore-R individuals from each developmental stage with Trizol (Invitrogen). For cellular localization of the TnaA proteins, soluble nuclear (SNF) and cytoplasmic fractions were obtained from Ore-R embryo collections of 3–21 hour postfertilization [15]. The SNF was also used for the TnaA coimmunoprecipitation (Co-IP) assays. For Osa Co-IP assays, a total soluble protein fraction was obtained from Ore-R embryo collections of 3–21 hour postfertilization [16]. Protein extracts from salivary glands of third instar larvae were obtained by collecting the glands in PBS buffer plus Complete protease inhibitors [EDTA-free protease inhibitor tablet (ROCHE)], and boiling them for 5 minutes in sample loading buffer. The proteins were separated by SDS-PAGE and electrotransfered to nitrocellulose membranes for Western blot analyses. Immunoblots were done according to standard procedures and proteins of interest were detected with specific antibodies using different chemoluminiscence kits (Supersignal West Pico Chemiluminescent Substrate from Thermo scientific, ECL Plus Western Blotting Detection System or ECL Advanced Western Blotting Detection kit from Amersham, GE Healthcare, USA), according to manufactureŕs instructions.

Affinity-purified primary TnaA_NH2_ and TnaA_XSPRING_ antibodies were used at a 1∶100 dilution. Anti-β-tubulin (E7, Developmental Studies Hybridoma Bank) and anti-Osa (Developmental Studies Hybridoma Bank) were used at 1∶3000 and 1∶1000 dilutions, respectively. The antibodies anti-Cdk7 (ds17, Santa Cruz), anti-RNA Pol II (8WG16, Covance) and anti-Hsp70 (ab2787, ABCAM) were used at 1∶1000, 1∶500 and 1∶600, respectively.

### Production and Affinity Purification of TnaA Antibodies

To generate antibodies against different TnaA regions, we used the TnaA cDNA that contains the TnaA translated exons from the ZAP1 clone [5] that represent the TnaA RD transcript [17]. The TnaA cDNA clone was digested with *Bam*HI and two fragments were independently subcloned into the pGEX2T vector to generate glutathione S-transferase (GST) fusion proteins harboring the TnaA amino-termini (amino acids 159–432, GST-TnaA_NH2–1_) and the XSPRING domain (amino acids 433–856, GST-TnaA_XSPRING1_). GST-fusion proteins were expressed and purified [18] to inject Winstar rats to raise polyclonal antibodies [19]. The antibodies from total sera were affinity-purified [20].

### Pull-down and Immunoprecipitation Assays

All the clones used in this work were nucleotide-sequenced. The *Drosophila* Ubc9 cDNA (BDGP Gold collection of *Drosophila* Genomics Resource Center) was amplified with the Forward: 5′-AGTTCGGAGAATTCTCCGGCATTGCTATTACACG-3′ and Reverse: 5′-CGGAATCCTCGAGGCG-CTTCTCGTACTCCAG-3′ primers, and cloned in the *Eco*RI and *Xho*I sites of the pGEX-4T vector. Pull-down assays were done as described previously [21]. Immunoprecipitations were done on SNF or total protein extracts from 3–21 hour postfertilization Ore-R embryos [22]. In these assays we made two preclearings steps and we used the Buffer PD (20 mM HEPES, pH 7.9, 100 mM NaCl, 1 mM EDTA, 4 mM MgCl_2_, 1 mM DTT, 0.1% NP-40, 10% glycerol and 0.2 mM PMSF).

### Yeast Two-hybrid Assays

*Drosophila* TnaA and Ubc9 cDNAs were cloned in the *Eco*RI and *Sal*I, and *Eco*RI and *Xho*I sites of the pGBKT7 and pGADT7 vectors, respectively. pGBKT7-TnaA was digested with *Bam*HI and religated to obtain the TnaA_NH2–2_ fragment (1–432 aa). TnaA_XSPRING2_ (379–927 aa) and TnaA_COO_ (929–1073 aa) were obtained after the digestion of the full-length pGBKT7-TnaA with *Nco*I and each fragment was cloned separately in *Nco*I-digested pGBKT7. TnaA_QLess_ (306–696 aa) and TnaA_SP-COO QLess_ (711–999 aa) were obtained by cloning in pGBKT7 digested with *Nde*I and *Eco*RI, PCR fragments obtained from the TnaA cDNA clone [5] using the primers: a) TnaA_QLess_ Forward 5′-GGAATTCCATATGCGACGAATGGC. CCCATATC-3′ and Reverse 5′-CGAGAATTCATCTG-GCCCGGCATTC-3′, b) TnaA_SP-COO QLess_ Forward 5′-GGACAGGCTCATATGGCCAAGATCTCATTGAAGTGC-3′ and Reverse 5′-GCAGAATTCCGTTT. GGGGCGAGTTGTG-3′. Osa_C2_ cDNA harbouring aminoacids 1951–2600 from the Osa protein was synthesized from polyA^+^ RNA from Ore-R embryos 3–21 hour postfertilization, according to [23]. The Osa_C2_ PCR fragment was synthesized with the Forward: 5′-GGAATTCTCCATATGAACTACACGATGGT. CACG-3′ and Reverse: 5′-CGTGAATTCCGTACCGCAGCTGTTGCTGTTG-3′ primers and cloned in pGADT7 with *Nde*I and *Eco*RI. Yeast two-hybrid assay was performed using the BD Matchmaker library construction and screening kit (Biosciences-Clontech). The interaction between baits and preys were tested evaluating the reporter genes *ADE2* and *HIS3* using the media QDO (SD-Trp/−Leu/−Ade/−His) supplemented with 3 mM 3-AT (3-amino-1,2,4-triazole).

### Fly Strains, Genetic Procedures, and Larval Staging

Unless otherwise noted, all mutations are described in Flybase [17]. Briefly, *tna^1^*, *tna^5^*, *osa^1^* and *osa^2^* are EMS-induced mutations. In *tna^1^* Gln 566 changed to a stop codon [5]. *tna^5^* was recovered after EMS mutagenesis in a genetic screen to identify *brm*-interacting mutations (J. A. K., unpublished results). The lesion in the *lwr^5^* allele (Arg 104 to His) is located in a region that has been involved in the interaction between ubiquitin-conjugating enzymes with the HECT or RING ubiquitin E3 ligases [24]. The *lwr^4–3^* and *lwr^13^* were both derived from imprecise excision of P-elements inserted in the 5′ regulatory zone [25], [26]. *smt3^04493^* is a P-element insertion 10 bp upstream of the first exon of *smt3* [27]. Fly cultures and crosses were performed according to standard procedures. Flies were raised on cornmeal-molasses media at 25°C unless otherwise noted. Media were supplemented with 0.05% of bromophenol blue to stage third instar larvae according to the gut dye clearance [28].

### Immunostaining of Ring Glands, Salivary Glands, and Polytene Chromosomes of Third Instar Larvae

Immunostaining of ring and salivary glands were done as described by [29], and the immunostaining of polytene salivary gland chromosomes was done as reported by [30]. For immunostaining of polytene salivary gland chromosomes, the TnaA_XSPRING_ antibodies were preabsorbed with fixed 0–3 hour embryos [31]. Polytene chromosomes and salivary and ring glands images were captured on a Leitz DMIRB inverted photoscope equipped with a Leica TCS Nt laser confocal imaging system, a Zeiss Inverted Axiovert fluorescent microscope, a Leica Aristaplan fluorescent microscope or an Olympus Inverted confocal FV1000 microscope. Images were processed using Image J.

## Results

### TnaA_130_ and TnaA_123_ in Space and Time throughout Development

**Figure 1:**
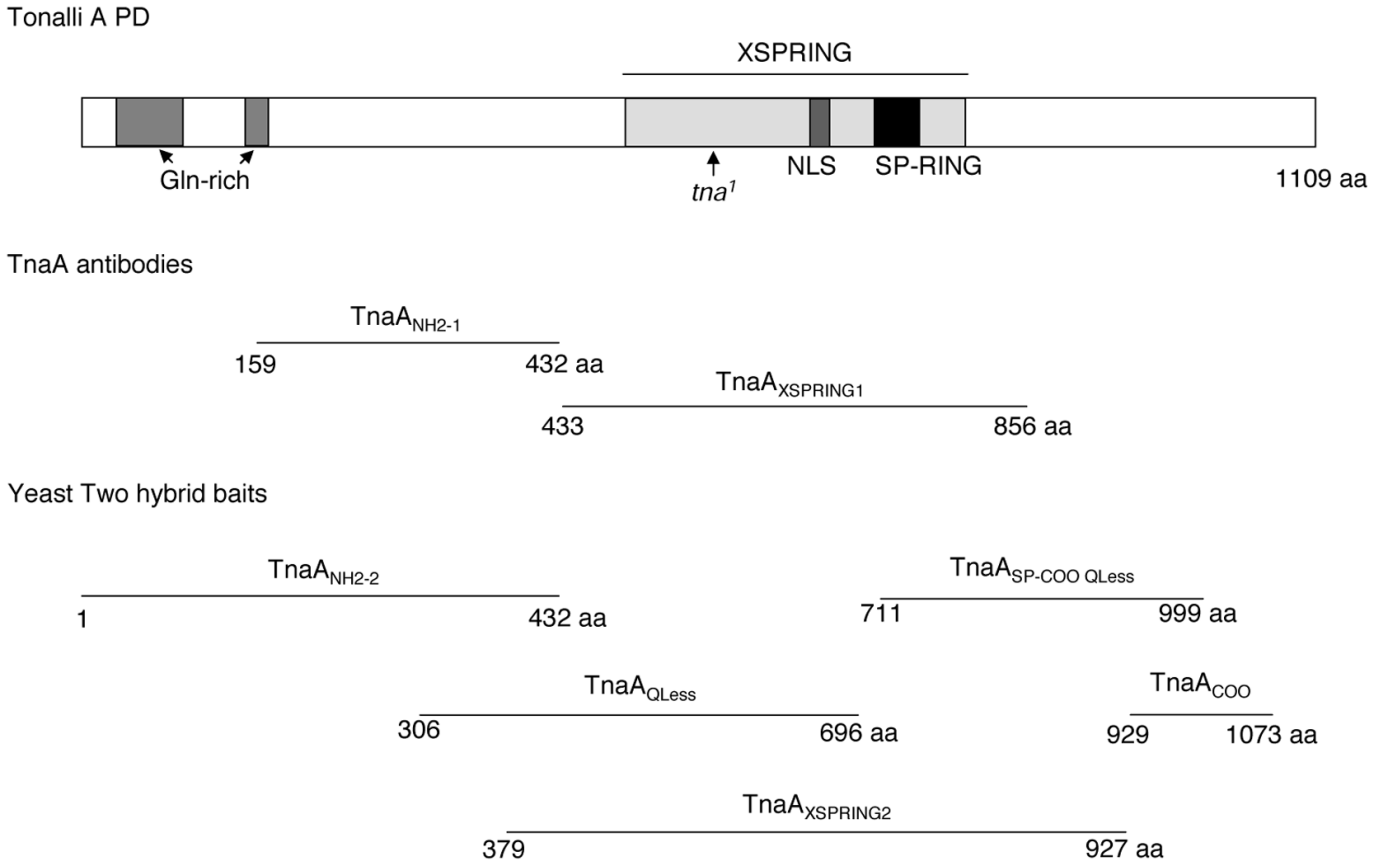
TnaA protein, domains and fragments. The TnaA protein (upper section). TnaA domains are indicated. Nuclear localization signal is NLS. The stop codon in the *tna^1^* allele [5] is indicated by an arrow. TnaA fragments used to produce TnaA antibodies from GST fusion proteins are shown (middle section). TnaA fragments fused to the yeast GAL4-binding domain to use as baits in two-hybrid assays (lower section).

**Figure 2:**
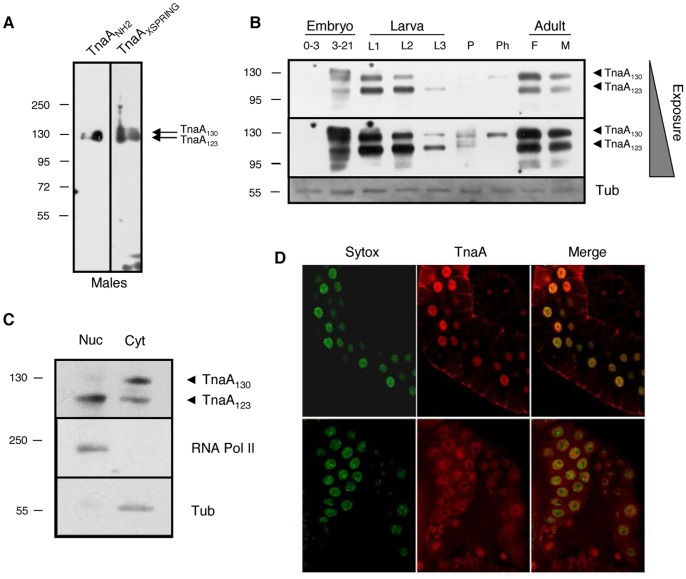
Expression and location of TnaA proteins throughout *Drosophila* development. (**A**) TnaA_NH2_ and TnaA_XSPRING_ antibodies detect the same proteins. TnaA proteins detected by full-range Western analysis in an adult male soluble protein extract with TnaA_NH2_ and TnaA_XSPRING_ antibodies (1∶100 dilution). (**B**) TnaA developmental Western. Detection of TnaA_130_ and Tna_123_ isoforms in soluble extracts isolated from embryos (0–3 and 3–21 hour), 1^st^, 2^nd^ and 3^rd^ instar larvae (L1, L2 and L3), pupae (P), pharate (Ph) and female and male adults (F and M). β-tubulin was used as a protein loading control (bottom). The TnaA_XSPRING_ and β-tubulin antibodies were used 1∶100 and 1∶1000, respectively. (**C**) TnaA_123_ is mainly nuclear. Detection of TnaA proteins in nuclear (Nuc) and cytoplasmic (Cyt) soluble fractions isolated from embryos 3–21 hour. The largest RNA polymerase II subunit and β-tubulin were used as controls of nuclear and cytoplasmic fractions, respectively. TnaA_NH2,_ RNA polymerase II, and β-tubulin antibodies were used 1∶120, 1∶500, and 1∶1000, respectively. (**D**) Immunostaining of TnaA in salivary (upper panel) and ring glands (lower panel) of Ore-R third instar larvae with TnaA_XSPRING_ (3∶5, red), DNA (Sytox, green) and merge (yellow). We detected no signal when immunostaining was done with secondary antibody only (not shown).

The *tna* gene produces several large transcripts that are differentially expressed from embryo through adult stages [5], [17]. The main large transcript is 6.1 kb and it peaks at the pupal stage [5]. Translation of this transcript predicts a protein product of 127 kDa that we named TnaA [5]. To study TnaA, we prepared two affinity-purified antibodies: TnaA_NH2_ that was raised against the amino-terminal region and TnaA_XSPRING_ that was raised against the XSPRING domain (Fig. 1 and Material and Methods). Both antibodies recognize the same proteins on adult male soluble extracts and they were used indistinctly along this work (Fig. 2A).

Two main TnaA protein products, one of 130 kDa (TnaA_130_) and another one of 123 kDa (TnaA_123_) are present in varying abundance throughout development (Fig. 2B). The abundance does not correspond to the *tna* mRNA expression pattern [5] suggesting postranscriptional regulation. We sometimes observe another product heavier than TnaA_130_ in embryos of 3–21 h (Fig. 2B). These three Tna species we found, are consistent with the three Tna polypeptides described in Flybase [17]. Nevertheless we cannot discard the possibility that TnaA could be postranslationally modified. For example, we determined using the SUMOsp 2.0 program [32] that TnaA has two putative SUMOylation sites and one putative SUMO Interacting Motif (SIM) [33] (data not shown). In extracts isolated from 0–3 hour embryos, we detected very low levels of TnaA_130_, while TnaA_123_ was not detected. In extracts isolated from 3–21 hour embryos, we detected a TnaA form larger than TnaA_130_, and the levels of both TnaA_130_ and TnaA_123_ increased, reaching maximums in the first larval instar. Decreases in the abundances of both proteins were observed in second and third instar larvae, with the levels of TnaA_123_ higher than those of TnaA_130_. Both forms abundance decreased substantially in pupae and TnaA_130_ was observed again at the pharate stage meanwhile TnaA_123_ is not detected. In adult flies of both sexes, TnaA_130_ and TnaA_123_ were both highly abundant at about equal levels. The appearance of TnaA_123_ was always preceded by the presence of TnaA_130_.

Next, we investigated the subcellular location of the TnaA proteins in nuclear and cytoplasmic fractions from 3–21 hour embryos (Fig. 2C, upper panel). The largest subunit of RNA polymerase II and β-tubulin were used to test the purity of the fractions (Fig. 2C, middle and lower panels). We found that TnaA_123_ was enriched in the nuclear fraction whereas TnaA_130_ was enriched in the cytoplasmic fraction (Fig. 2C). It has been shown that SUMO is present in prothoracic gland nuclei [29] in third instar larvae. *tna* mutant individuals arrest development at the larval-pupal transition which is where less TnaA protein is expressed (see ahead). This suggests that TnaA may be expressed in specific tissues relevant for metamorphosis. We immunostained salivary (Fig. 2D, upper panel) and ring glands (Fig. 2D, lower panel) from third instar larvae with the TnaA_XSPRING_ antibody and we found that TnaA was present most highly within the nucleus of the secretory cells of salivary glands and in prothoracic gland cells.

### TnaA is Critical for Larval Development

**Figure 3:**
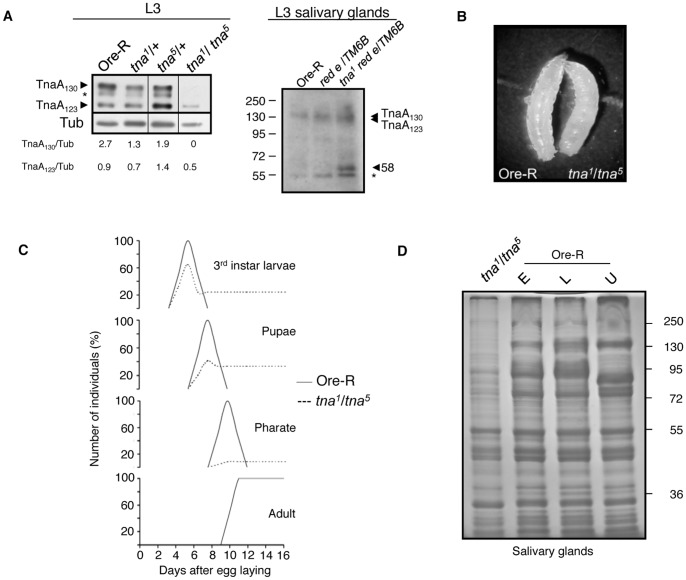
Characterization of *tna^1^*/*tna^5^* individuals. (**A**) TnaA proteins in *tna^1^*/*tna^5^* third instar larvae (left panel). Note the absence of TnaA_130_ and the reduction of TnaA_123_ levels. Western blot probed with the TnaA_XSPRING_ antibody (1∶100) was done with similar amount of proteins from total third instar larvae extracts of the indicated genotype. The band marked with an asterisk (*) appears sometimes depending on extracts and running gel conditions and may represent a processed protein product. Narrow vertical lines indicate edition of lanes from the same film with samples from flies with other *tna* genotypes not relevant to this work. β-tubulin was used as loading control (antibody used 1∶1000). The levels of TnaA_130_ and TnaA_123_ were compared with the level of β-tubulin using Image-J. Western blot of *tna^1^ red e^/^TM6B* salivary gland extracts probed with TnaA_NH2_ antibody (right panel) detected TnaA_130_, TnaA_123_ (that comigrated in this gel) and a 58 kDa band close to the predicted size of a truncated Tna-1 protein (62 kDa). Salivary gland extracts from OreR individuals and from individuals harbouring the *tna^1^* parental (*red e*) and balancer (*TM6B*) chromosomes were used as controls. The asterisk indicates a crossreacting band present in all the genotypes tested. (**B**) *tna^1^*/*tna^5^* and Ore-R third instar larvae. (**C**) Survival percentage of *tna^1^*/*tna^5^* individuals at different stages of development. Ore-R (−) and *tna^1^/tna^5^* (–) survival percentages are indicated. More than 100 third instar larvae were counted for each genotype. Heterozygous *tna* individuals have survival rates similar to those for Ore-R. (**D**) Protein profile of *tna^1^*/*tna^5^* salivary glands. Note the differences between the *tna^1^*/*tna^5^* and the Ore-R total protein profiles. SDS-PAGE of soluble protein extracts from 10 pairs of larval salivary glands stained with Coomasie blue. Ore-R salivary glands extracts from early (E) and late (L) or unstaged (U) third instar larvae that were loaded as references.

While we can detect TnaA_130_ and TnaA_123_ in Ore-R and in *tna^1^/*+ or *tna^5^/*+ individuals, TnaA_130_ is barely detectable and TnaA_123_ decreases dramatically in *tna^1^*/*tna^5^* larvae (Fig. 3A, left panel). The *tna^1^* mutation changes Gln 566 to a stop codon, is recessive lethal [5] and behaves as a dominant negative. *tna^1^* is a much stronger dominant enchancer of *osa^1^* than is a deficiency of the *tna* region (Table 1). *tna^1^* would produce a truncated protein of 62 kDa that we have been able to observe in heterozygous *tna^1^/+* salivary glands soluble extracts (Fig. 3A, right panel). The molecular lesion of *tna^5^* has not been determined, but it behaves genetically as a hypomorphic allele and its product can be detected in *tna^1^/tna^5^* third instar larvae extracts (Fig. 3A, left panel).

**Table 1:** Genetic interactions of *tna* and *osa* with SUMOylation pathway genes.

Genotype	Number of flies with HWO^a^	Penetrance (%)^a^
+/*osa^1^*	9/265	3
+/*osa^2^*	0/303	0
+/*smt3^04493^*	0/389	0
+/*lwr^4–3^*	1/133	1
+/*lwr^5^*	0/297	0
+/*lwr^13^*	0/341	0
+/*tna^1^*	116/624	19
*tna^1^*/*osa^1^*	327/334	98
*Df(3L)lxd6*/*osa^1^*	16/115	14
*tna^1^*/*osa^2^*	140/334	42
*^b^smt3^04493^*/+; *tna^1^*/+	44/249	18
*^c^smt3^04493^*/+; *tna^1^*/+	66/118	56
*lwr^4–3^*/+; *tna^1^*/+	69/150	46
*lwr^5^*/+; *tna^1^*/+	71/282	25
*lwr^13^*/+; *tna^1^*/+	177/343	52
*smt3^04493^*/+; *osa^1^*/+	57/264	22
*smt3^04493^*/+; *osa^2^*/+	0/216	0
*lwr^5^*/+; *osa^1^*/+	1/147	1
*lwr^13^*/+; *osa^1^*/+	0/124	0
*lwr^5^*/+; *osa^2^*/+	0/212	0
*lwr^13^*/+; *osa^2^*/+	0/256	0

a For expressivity of held-out wing phenotype (HWO) see Fig. 6.

b, c Reciprocal crosses were done were done in all cases with no observed differences except in the crosses with *tna^1^* males and *smt3^04493^* females^b^, or with *smt3^04493^* males and *tna^1^* females^c^. At least 100 flies were examined for each genotype. Flies that do not present the held-out wing phenotype include *tna^3^, tna^5^*, or *tna^−^* deficiencies *Df(3L)vin2* and *Df(3L)lxd6*, *osa^2^*, *lwr^5^*, *lwr^4–3^*, *lwr^13^*, *smt3^04493^* heterozygous individuals, *lwr/+*;*osa/+*, *smt3^04493^*/+;*osa^2^*/+ and all the transheterozygous combinations between *tna^3^, tna^5^*, or *Df(3L)vin2* and *Df(3L)lxd6* with *smt3* and *lwr* alleles.

To better understand *tna* function we studied the lethality of *tna^1^/tna^5^* animals. The *tna^1^*/*tna^5^* larvae (Fig. 3B) did not have melanotic tumors as observed in *lwr* or *aos1* mutant individuals [34], [35], [36], nor are they a larger size as observed for *smt3* knockdowned larvae [29]. We found that 65% of *tna^1^*/*tna^5^* individuals reach the third instar larval stage (Fig. 3C), but only 41% pupated and only 8% of the expected individuals reached the pharate stage. No *tna^1^/tna^5^* individuals eclosed as adults (Fig. 3C). We also noticed that the *tna^1^/tna^5^* third instar larvae that did not pupate often survived long after their heterozygous *tna^1^/+* or *tna^5^/+* siblings larvae pupated. Some of these *tna^1^*/*tna^5^* larvae have an extended lifespan of at least two weeks (Fig. 3C). A similar extension of larval lifespan was previously observed in animals with reduced levels of SUMO [29], Aos1 (one of the E1 subunits) [34] or Ubc9 (E2) [35], [36].

Given the abnormal behavior of *tna^1^*/*tna^5^* larvae and knowing that the TnaA profile is altered (Fig. 3A), we characterized the protein profile of their salivary glands (Fig. 3D). We staged the larvae by feeding them with bromophenol blue [28] and divided them in early (blue) and late (white) larvae. All *tna^1^*/*tna^5^* larvae remained as early larvae (blue). They were collected 24 hours after they crawled from the food to obtain their salivary glands and we determined their protein profile (Fig. 3D). Although *tna^1^*/*tna^5^* larvae remained blue, the protein profile differed from both the early and late wild-type Ore-R salivary glands obtained under the same conditions. Differences in the quantity and quality of proteins present in *tna^1^*/*tna^5^* salivary glands fall mostly in the range over 72 kDa (Fig. 3D).

### TnaA is Chromatin-associated at Discrete Sites on Polytene Salivary Gland Chromosomes

**Figure 4:**
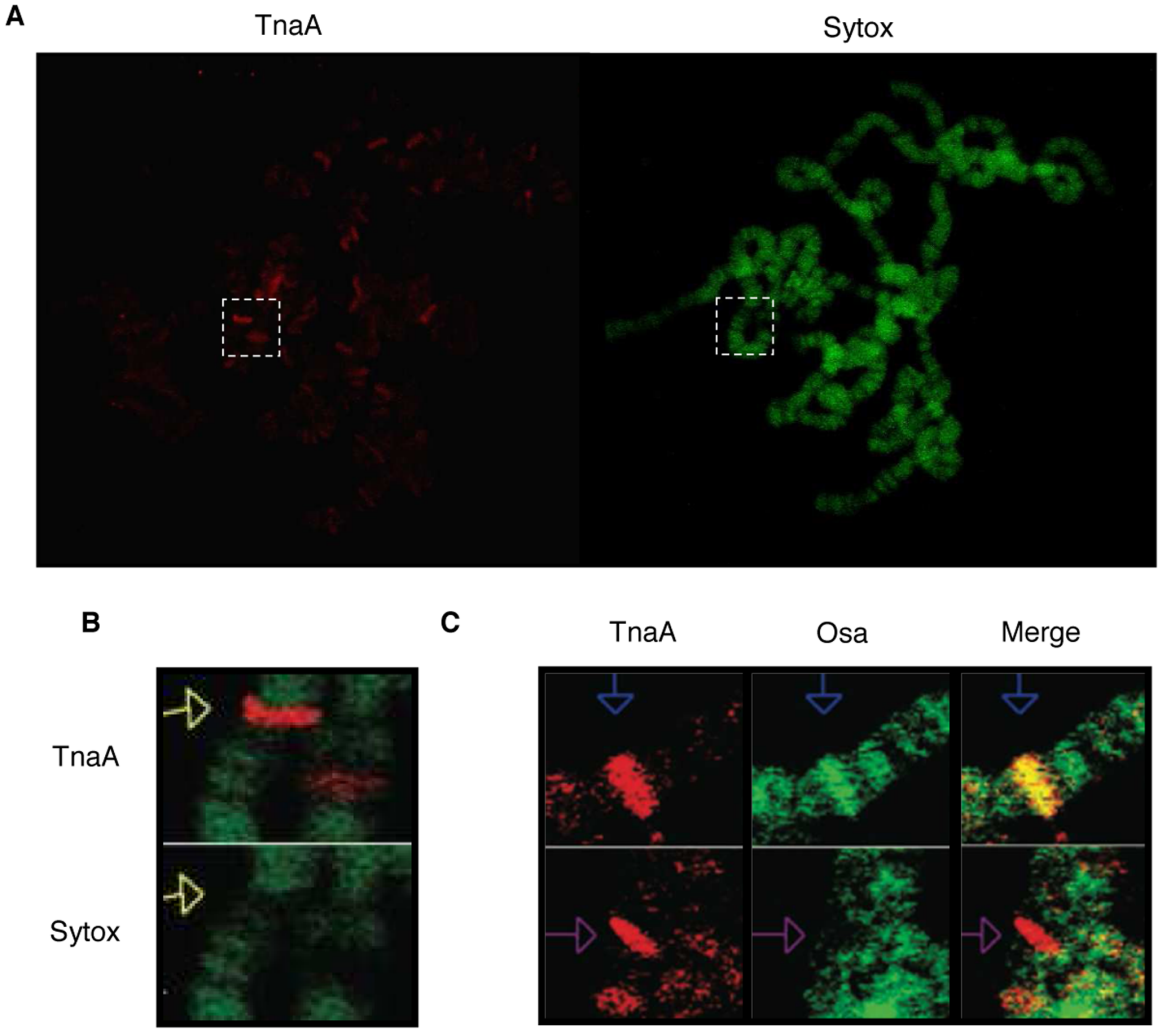
TnaA is located on polytene salivary gland chromosomes of third instar larvae and sometimes colocalizes with Osa. (**A**) Immunostaining of TnaA in Ore-R (wild type) polytene salivary gland chromosomes of third instar larvae. TnaA_XSPRING_ antibody (1∶50, red) and DNA (Sytox, green). Amplification in B is indicated (pointed white rectangle). (**B**) TnaA is located in chromatin interbands. (**C**) TnaA and Osa colocalize in some sites on polytene salivary gland chromosomes of third instar larvae (blue arrows in the top panels) but in others do not (purple arrows in the bottom panels). TnaA_XSPRING_ antibody (1∶50, red) and Osa (1∶50, green). No signal was detected when no primary antibody was added (data not shown).

We have shown that TnaA_123_ is nuclear in *Drosophila* embryos (Fig. 2C) and that TnaA (probably TnaA_123_) is mainly nuclear in salivary and ring glands from third instar larvae (Fig. 2D). We immunostained polytene salivary gland chromosomes of third instar larvae and found that TnaA is associated with discrete sites (Fig. 4A). The number of TnaA sites suggests that TnaA might be required for the transcription of more than just the homeotic genes. Interestingly, most of the TnaA signals detected on polytene salivary gland chromosomes are located in interbands which are thought to have decondensed chromatin where transcription can occur (Fig. 4B). Because of the strong genetic interactions between *tna* and *osa* [5], we coimmunostained for TnaA and Osa on polytene salivary gland chromosomes. TnaA colocalizes with Osa at some sites, but not at others (Fig. 4C, upper and bottom panels). We do not know whether this is because TnaA is not required at all genes regulated by Osa, or whether it is due to an interaction between TnaA and Osa that is more transient than Osa localization.

### TnaA Physically Interacts with Ubc9 and with Osa

SUMO E3 ligases function for selection of SUMOylation targets and/or for enhancement of the SUMO conjugation process. TnaA has an SP-RING zinc finger that is also present in a subclass of SUMO E3 ligases that includes the PIAS proteins in mammals [37] and Su(var)2–10 in *Drosophila* [14]. Since the SP-RING in the PIAS proteins physically interacts with Ubc9 [38], [39], we explored whether TnaA physically interacts with *Drosophila* Ubc9, using yeast two-hybrid assays and pull-down assays.

**Figure 5:**
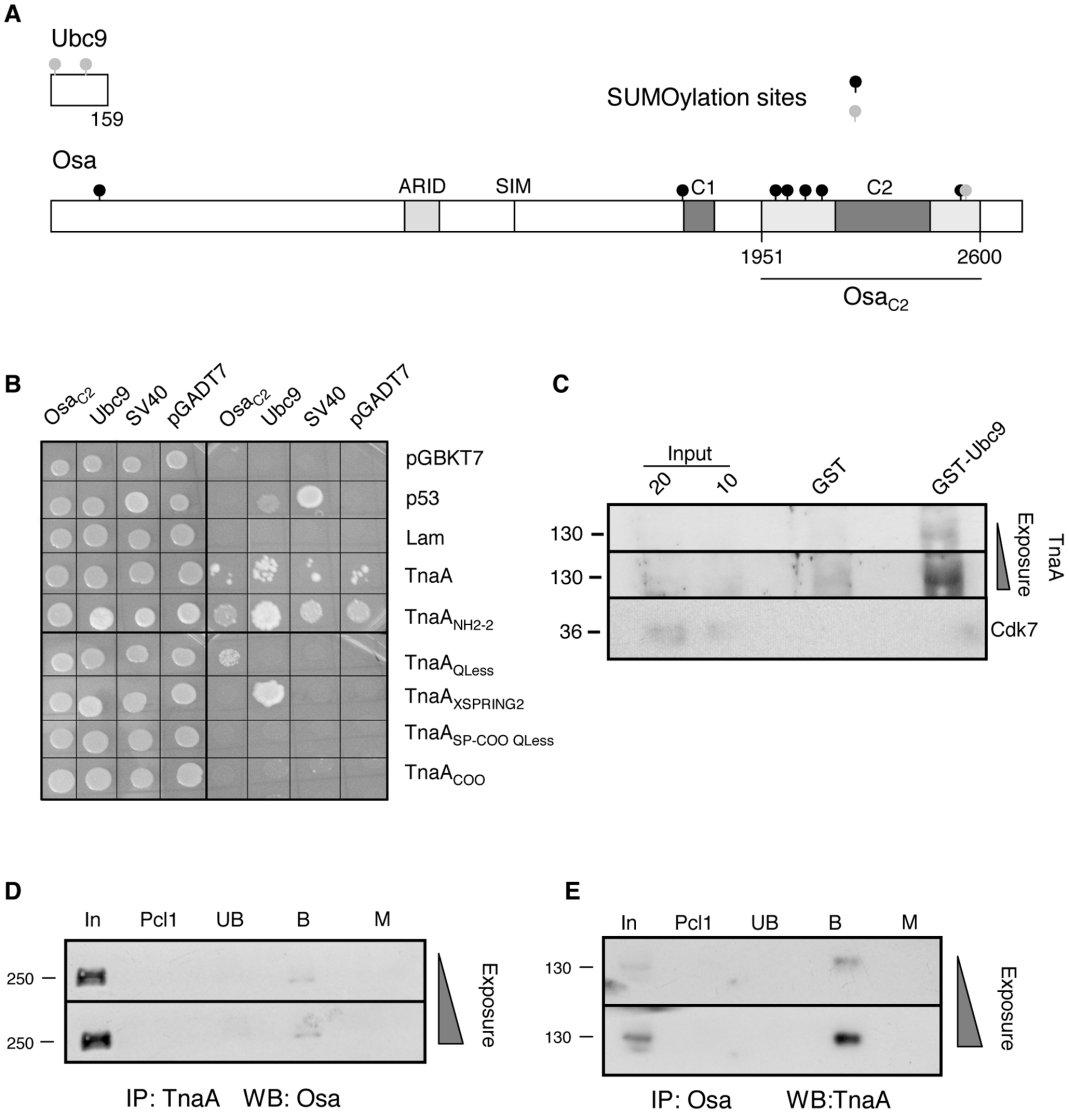
TnaA interacts with *Drosophila* Ubc9 and with Osa. (**A**) Schemes of *Drosophila* Ubc9 and Osa_C2_ used in biochemical assays. In the Osa protein, the ARID, the C1 and C2 domains (grey boxes), the SUMO interacting motif (SIM) and the Osa_C2_ fragment (dark line) are indicated. Forward (black circles) and inverted (gray circles) putative SUMOylation consensus sites in these proteins are indicated. For TnaA baits see Fig. 1. (**B**) TnaA interaction with Ubc9 and Osa_C2_ in yeast two-hybrid assays. Yeast colony complementation of growth controls in SD-Trp/−Leu media due to the presence of pGBKT7 (Trp^+^) and pGADT7, (Leu^+^) plasmids (left) in the same yeast cells. Interaction assay in QDO +3-AT (SD-Trp/−Leu/−Ade/−His +3-AT) media (right). Growth is observed when baits and preys interact, allowing GAL4 reconstitution with the consequent *ADE2* and *HIS3* reporter genes transcription. Baits were TnaA fragments (Fig. 1) fused to the DNA-binding domain of GAL4 in pGBKT7. Ubc9 and Osa_C2_ were preys fused to the GAL4 activation domain in pGADT7. Human p53 (p53) and Lamin C (Lam) interactions with SV40 are positive and negative controls, respectively. (**C**) TnaA interaction with Ubc9 by pull-down. The assays were done with 10 µg of each GST or GST-Ubc9 as baits and with 500 µg of soluble nuclear fraction from 3–21 hour embryos. 10 and 20% of the extract are shown as Input. TnaA was detected by Western analysis with TnaA_XSPRING_ antibody (1∶100) when GST-Ubc9 was used as bait. The 130 kDa weight marker is indicated (left) and increasing exposures of the same membrane are shown. Cdk7 was detected only in the Input lanes (antibody dilution, 1∶1000). (**D**) Coimmunoprecipitation of Osa with TnaA antibodies from nuclear extracts obtained from 3–21 hour embryos. TnaA_XSPRING_ antibodies (1 µg), and 3–21 hour embryos soluble nuclear fraction (500 µg) were used. The Western was revealed with the Osa antibody (1∶1000). Input, (In), preclearing 1 (Pcl1), unbound (Ub), bound (B). Immunoprecipitation with the equivalent amount of a preimmune serum instead of TnaA_XSPRING_ antibody was used as Mock (M). Both panels show films with increasing exposure time of the same membrane. (**E**) Coimmunoprecipitation of TnaA with Osa antibodies from total extracts obtained from 3–21 hour embryos. Osa antibodies (1 µg), and 3–21 hour embryos soluble nuclear fraction (3.7 mg) were used. The Western was revealed with the TnaA_NH2_ antibody (1∶120). Lanes are labeled as above. The equivalent amount of an irrelevant antibody was used as mock (M). Molecular weight markers are indicated (left).

For the yeast two-hybrid assays we first used the full-length TnaA protein (Fig. 1) fused to the yeast GAL4-DNA binding domain as “bait”, and the full-length *Drosophila* Ubc9 protein (Fig. 5A) fused to the GAL4-activation domain as “prey”. We found that the full-length TnaA protein was able to activate the transcription of at least two reporter genes in the absence of a “prey” (Fig. 5B), and as a consequence the full-length TnaA protein could not be used to test for the Ubc9 interaction in this assay. We then split the TnaA protein into five fragments that cover the whole TnaA protein (Fig. 1). Two out of the five fragments contain the SP-RING zinc finger (TnaA_XSPRING2_ and TnaA_SP-COO Qless_). The other fragments have different TnaA regions that include the two glutamine-rich domains (TnaA_NH2–2_), the bipartite nuclear location signal (TnaA_QLess_) and the carboxy-ending (TnaA_COO_). We found that the TnaA_XSPRING2_ fragment interacted with Ubc9 in the yeast two-hybrid assay while the other fragments, including TnaA_SP-COO QLess_, did not interact (Fig. 5B). These results show that the TnaA SP-RING zinc finger is necessary but not sufficient for the TnaA interaction with Ubc9 in this assay.

Osa is a subunit of some BRM complexes, and the *osa* gene strongly interacts with *tna* [5]. Since it was found that Osa is modified by SUMO in *Drosophila* embryos [3], we thought that TnaA might be involved in Osa SUMOylation. We searched for SUMOylation consensus sites (ψKxE) in the Osa protein sequence (2713 aa) using the SUMOsp 2.0 program [32] and found eight putative SUMOylation sites (Fig. 5A), six of them located within a segment located from amino acids 1951 to 2600 surrounding the C2 domain [40]. We will refer to the fragment with the six putative SUMOylation sites as Osa_C2_ in this work. We synthesized the Osa_C2_ cDNA from polyA^+^ RNA of 3–21 hour embryos and fused it to the GAL4-activation domain to use as “prey” in the yeast two-hybrid assay. We tested the six TnaA baits already described (including full-length TnaA), and found that baits harbouring the SP-RING (TnaA_XSPRING2_ or TnaA_SP-COO QLess_) did not interact with the Osa_C2_ prey. Although TnaA_NH2–2_ (and to a lesser extent, full-length TnaA) interacted with Osa_C2_, these baits also interacted with pGADT7 or pGADT7-SV40 negative control samples, preventing us from concluding whether the interactions with Osa_C2_ are bona fide. In contrast, we found that the TnaA_Qless_ bait cleanly interacts physically with Osa_C2_ (Fig. 5B).

Although the TnaA_XSPRING2_ region interacted physically with Ubc9 in the yeast two-hybrid assays, we wanted to test for TnaA/Ubc9 physical interactions in *Drosophila* embryos. We performed pull-down assays using as bait a purified GST-Ubc9 fusion protein incubated with a nuclear protein extract from 3–21 hour embryos where we know TnaA is present (Fig. 2B). After extensive stringent washing, the presence of TnaA amongst the GST-Ubc9-interacting proteins was assessed by Western analyses with the TnaA_XSPRING_ antibody (Fig. 5C). As expected, we found that full-length TnaA from nuclei of *Drosophila* embryos interacts with full length GST-Ubc9, confirming the results that we obtained with the yeast two-hybrid assays using TnaA fragments and further suggesting that these proteins interact *in vivo*.

In all reported cases it is known that only a fraction of the whole pool of a SUMOylatable protein in a cell is SUMOylated, either because of spatial restrictions (the target protein should be located where the SUMO and the SUMOylation enzymes are) or because fine regulation constricts the amount of the SUMOylated protein [4]. We showed that Osa_C2_ interacts with a fragment of TnaA (TnaA_QLess_) in a yeast two-hybrid assay (Fig. 5B). To test whether this interaction can be observed with the full-length proteins in *Drosophila* embryos, we performed TnaA or Osa coimmunoprecipitation assays from total or nuclear protein extracts from 3–21 hour embryos. For this purpose, we first showed that the TnaA_XSPRING_ and Osa antibodies are able to immunoprecipitate TnaA and Osa, respectively (Fig. S1), and that the control proteins Hsp70 and Cdk7 do not coimmunoprecipitate with TnaA or with Osa, respectively (Fig. S2). Interestingly, we found that TnaA coimmunoprecipitates with a fraction of Osa found in nuclear protein extracts from 3–21 hour embryos (Fig. 5D), and that reciprocally, Osa coimmunoprecipitates with TnaA from a total protein extract of 3–21 hour embryos (Fig. 5E). Since we found that TnaA interacts physically with Osa and with Ubc9 (Fig. 5) we tried to test whether TnaA has SUMO E3 ligase activity on the Osa_C2_ fragment using a mammalian *in vitro* assay (Active Motif kit). Although Osa_C2_ is SUMOylated in this assay, we were not able to get convincing evidence that TnaA has SUMO E3 ligase activity under these conditions (data not shown).

### *tna* and *osa* Genetically Interact with Components of the SUMOylation Pathway

**Figure 6:**
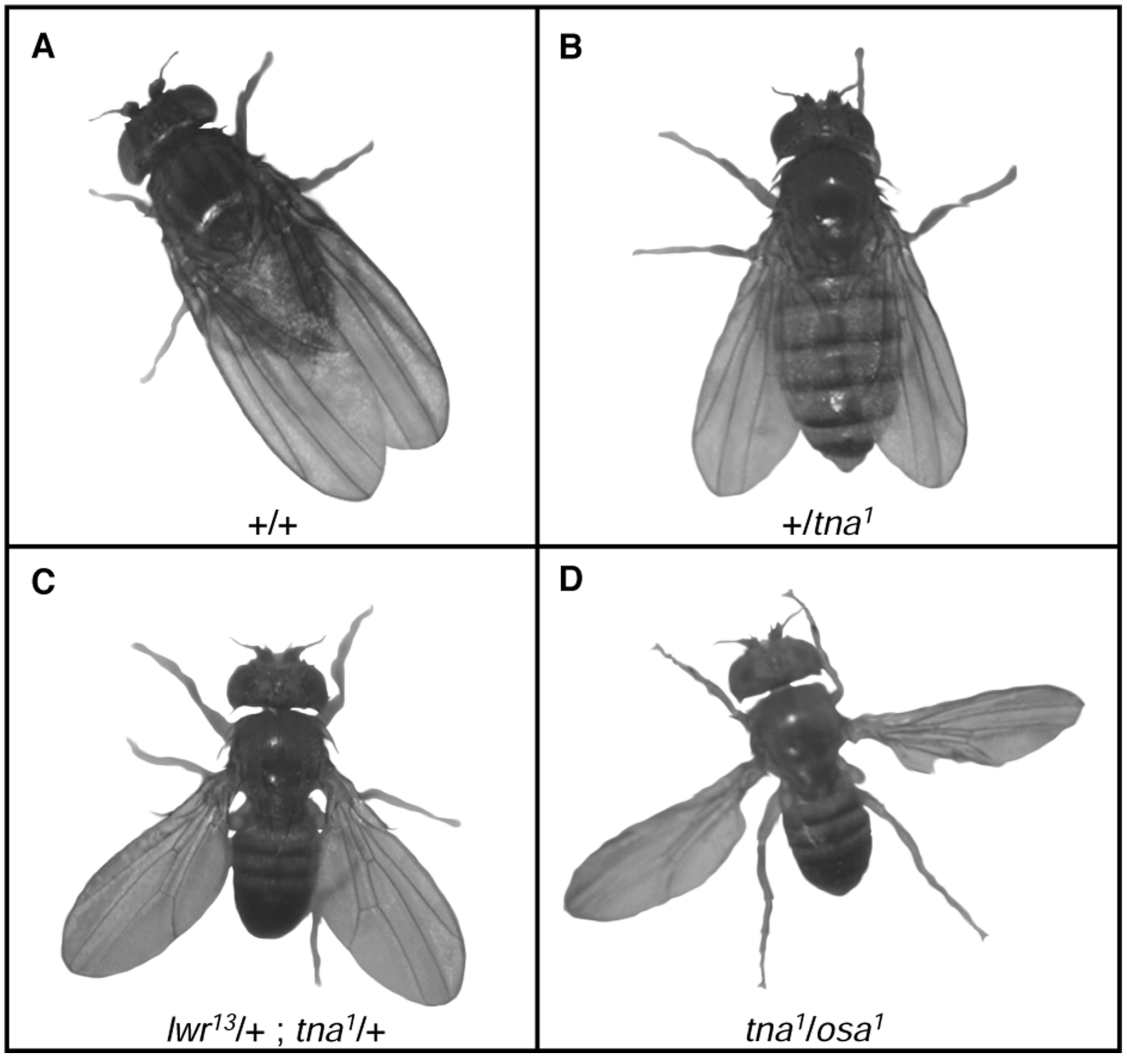
SUMOylation pathway mutations enhance held-out wing phenotype of *tna* and *osa* flies. Flies with different held-out wing phenotype expressivity. Fly genotype is indicated in each picture. Penetrance of the held-out wing phenotype in each genotype is in Table 1 (**A**) Wild type fly (**B**) Slight held-out wing phenotype of +/*tna^1^* flies. The same phenotype is presented by +/*osa^1^* individuals. (**C**) Stronger held-out wing phenotype of *smt3/*+;*tna^1^/*+ individuals. The same phenotype is presented by *lwr/*+;*tna^1^/*+ or *smt3^04493^*/+;*osa^1^*/+ individuals. (**D**) Strongest held-out wing phenotype of *tna^1^*/*osa^1^* individuals. This phenotype is also presented by *tna^1^*/*osa^2^* individuals.

*tna* genetically interacts with *brm* and *osa* [5]. Transheterozygous adult flies carrying mutations in combinations of any of these three genes have a strong held-out wing phenotype [5], [8] (Fig. 6). This phenotype appears to result from reduced expression from the P2 promoter of the homeotic gene *Antp* [8]. The interactions with *tna* might be a consequence of reduced SUMOylation of Osa (and/or Brm) proteins. If so, mutations in other components of the SUMOylation pathway might also show genetic interactions. We generated transheterozygous flies carrying mutant alleles of either the SUMO E2 conjugating enzyme Ubc9 (*lwr^5^*, *lwr^4–3^*, and *lwr^13^*) [25], [26] or SUMO (*smt3^04493^*) [27] in combination with mutant alleles of *tna* (*tna^1^* and *tna^5^*) or *osa* (*osa^1^* and *osa^2^*). All of these individuals have at least one wild type copy of each gene to allow survival to the adult stage.

Individuals carrying *tna* alleles other than *tna^1^* (*tna^3^* or *tna^5^*), or deficiencies uncovering the *tna* region [*Df(3L)vin2* or *Df(3L)lxd6*] do not show the held-out wing phenotype. This phenotype is also not shown by transheterozygous individuals carrying *tna* alleles other than *tna^1^* or the *tna* deficiencies in combination with *smt3* or *lwr* alleles. In contrast, we found that *smt3^04493^*, *lwr^4–3^* and *lwr* ^13^ enhance both the penetrance and the expressivity of the held-out wing phenotype of *tna^1^* individuals (from 19% with weak expressivity to 56%, 46% and 52% with stronger expressivity, respectively, Fig. 6 and Table 1). Thus, we confirmed genetically that *tna* interacts with the SUMOylation pathway genes *smt3* and *lwr*. Interestingly, we found a maternal effect in the enhancement of the *tna^1^* held-out wings phenotype in double heterozygous *smt3^04493^*/+; *tna^1^*/+ individuals. The enhancement is only observed when there is not maternal contribution of *tna* (Table 1). We did not observe this maternal effect with the other genes tested. Since we observed strong genetic interactions between *tna* and *osa* (Fig. 6) [5], therefore we tested whether the SUMOylation genes *lwr* and *smt3* also interact with *osa* (*osa^1^* and *osa^2^* in Table 1 and Fig. 6). We found that *smt3^04493^* augmented the penetrance and the expressivity of the weak held-out wing phenotype of *osa^1^* (Fig. 6, Table 1) but that it did not interact with the weaker *osa^2^* allele. None of the *lwr* alleles tested interacted with *osa^1^* or *osa^2^*, suggesting that Ubc9 activity in these heterozygous individuals is sufficient to reach appropriate SUMOylation levels.

## Discussion

The presence of the SP-RING, the physical interaction of the SUMO E2 conjugating enzyme Ubc9 with TnaA, and the genetic interaction of *tna* with genes encoding SUMOylation pathway proteins suggest that TnaA may be involved in the SUMOylation pathway to activate transcription. TnaA may also have other functions not directly related to SUMOylation. These other functions may or may not act together with SUMOylation to positively regulate gene expression.

### TnaA Function in Gene Expression Involving the SUMOylation Pathway

Gene expression involves the integration of many regulatory mechanisms. Recently, many examples of SUMOylation and/or ubiquitylation during transcriptional regulation have been described [4]. These examples include the clearance of activators to favor transcription cycles in inducible genes [41] and the assembly of different proteins into a complex [42], [43]. Most of the *tna* interacting genes (*osa*, *brahma*, *moira*, *kohtalo*, *skuld*, and *kismet*) [5] encode subunits of complexes involved in chromatin remodeling and transcription by RNA polymerase II, suggesting that SUMOylation may be important at multiple aspects of gene regulation in *Drosophila*. Typically, SUMO-tagged proteins are recognized by a binding partner that contains a SIM (SUMO Interacting Motif) [33]. All of the proteins encoded by the *tna* interacting genes listed above have more than one SIM and SUMOylation sites (data not shown) and could be either SUMOylation targets, readers of the SUMO mark, or proteins that help TnaA exert its function(s).

SUMO E3 ligases are required for the enhancement and/or for the specifity of the SUMOylation tagging on targets. In this work we utilized different approaches to show that TnaA is involved in the SUMOylation pathway possibly as a SUMO E3 ligase. We showed a TnaA physical interaction with Ubc9 and genetic interactions between *tna* and *osa* with SUMOylation pathway genes. SUMOylated Osa is found in early embryos (0–3 hour) [3] and embryonic TnaA and Osa coimmunoprecipitate reciprocally (this work). We also showed that a GST-Ubc9 fusion physically interacts with native nuclear TnaA from *Drosophila* embryos. Hence, we suggest that Osa is a good candidate to be a TnaA-SUMOylation target *in vivo*. Our data suggest that TnaA-dependent SUMOylation of Osa and/or of other target(s), particularly proteins associated with Osa (e.g. other BRM complex subunits, histones, or others, see ahead), may be required for correct gene expression including homeotic genes. Osa is a large protein of around 280 kDa with an ARID domain which binds AT-rich sequences, LXXLL domains [8] that could help it to interact with nuclear receptors and has eight putative SUMOylation target sequences, six of them in the Osa_C2_ fragment (Fig. 5A). In humans there are three proteins related to Osa, BAF250a, BAF250b and BAF200/ARID2 [44] and it was reported that BAF250b could be in a complex that has E3 ubiquitin ligase activity on histone H2B [45].

Originally *tna* was identified in a screen to find Brm-interacting proteins [5]. Although we did not study here whether Brm can be SUMOylated, it has been reported that mammalian SUMO-2 can be acetylated at K33 to inhibit some SUMO-SIM interactions [46]. Interestingly, these authors also show that the bromodomain of p300, besides recognizing acetylated histones [47], can bind the SUMO acetylated form, opening the question of whether other bromodomains, such as the one present in the Brahma protein, would be able to recognize a putative *Drosophila* acetylated SUMO when present in any of its interactor proteins.

TnaA may also be promoting homeotic gene expression by inactivation through SUMOylation of a PcG protein. Indeed, SUMOylation of the PcG protein Scm (encoded by the *Sex comb on midleg* gene) decreases its levels at the PRE (Polycomb Response Element) located upstream the *Ubx* homeotic gene. SUMO compromised animals show a reduction of *Ubx* expression and it has been suggested that TnaA may be involved in Scm SUMOylation to promote homeotic gene expression [48].

### Other TnaA Interactors and SUMO-independent Functions of TnaA

We found that TnaA_130_ is mainly cytoplasmic and TnaA_123_ is mainly nuclear. Although most studied SUMO enzymes and targets are in the nucleus, there are some examples of SUMOylation of proteins in the cytoplasm [49]. As TnaA_130_ always precedes the appearance of TnaA_123_ through development (developmental Western, Fig. 2B), we think that TnaA may be processed to enter the nucleus to SUMOylate its targets. Notably, SUMOylation pathway proteins with well known nuclear activities also SUMOylate targets in the cytoplasm [50]. Thus, with what we know at present, we cannot discard the possibility that TnaA_130_ can also function in the cytoplasm. We also found that *tna* interacts with the *γTub23C* gene that encodes an isoform of γ-tubulin [51] and with *taranis* (*tara*) [5]. The significance of the interaction of *tna* with *tara* and *γTub23C* is currently unknown.

It is possible that TnaA could be necessary for BRM complex(es) function(s) regardless of SUMOylation, and that independently, SUMOylation could be required for function of other BRM complex(es) components. We cannot neither rule out the possibility that TnaA may have other functions independent of its possible role in the SUMOylation pathway, as has been reported for the PIAS proteins, known SP-RING SUMO E3 ligases [52], [53], [54]. The SP-RING plays a key role in this PIAS activity. The TnaA SP-RING is immersed in a 300-aminoacid region that we called the XSPRING domain that is shared with the vertebrate proteins Zimp7 and Zimp10 [KIAA1886 and KIAA1224 respectively, 5]. Although TnaA is related to the PIAS proteins because it has an SP-RING, it does not have the SAP (Scaffold attachment factor-A/B, Acinus and PIAS domain) nor the PINIT motifs that are PIAS signature domains.

The SAP and PINIT motifs in the PIAS proteins confer functions related to structural anchoring and transcriptional regulation. In mammals it has been shown that PIAS1 promotes the transcriptional repressive activity of Msx1 through regulating its location in a SUMO-independent way [53], it controls the stability of Msx1 by preventing its ubiquitination [55] and it regulates the transcriptional activity of GATA4 [56]. Similarly, in *Xenopus*, XPIASy down-regulate XSmad2 transcriptional activity independently from XPIASy SUMO E3 ligase activity [57].

Although human Zimp7, human Zimp10, and *Drosophila* TnaA do not have these other PIAS signature motifs they have transcriptional activation domains [58], [59] (Fig. 5B). The presence of a transcriptional activation domain could explain why we could not use the TnaA-Gal4 DNA-binding domain fusion in the yeast two-hybrid system (Fig. 5B). This suggests that TnaA, besides its possible role in the SUMOylation pathway, has other functions in *Drosophila* transcriptional activation.

### TnaA in *Drosophila* Development

We described a genetic interaction between *tna*, *osa*, and SUMOylation pathway genes. TnaA interacts physically with Ubc9 through the SP-RING supporting the genetic interaction data. Animals derived from *osa* and *tna* mutant germline clones die at different stages of development. While the *osa* ones do not survive embryogenesis [8] the *tna* ones die mostly as third instar larvae [5]. A pool of Osa is found SUMOylated in embryos of 0–3 hour of development when zygotic expression has not started [3] and TnaA is barely detectable (overexposure of Fig. 2B, data not shown). Moreover, when we studied the *tna* and *smt3* interaction, we found a *tna* maternal effect. The held-out wings phenotype in *smt3*/+; *tna*/+ adults is observed when the mother is *tna* defective, but we do not observe this when the mothers have low dosages of SUMO (Table 1). We think it is probable that SUMOylated Osa plays a role at early stages of development. SUMOylation of embryonic Osa can happen in the maternal germline or in the embryo with the help of the maternally-inherited SUMOylation pathway machinery. This machinery may include TnaA if TnaA is involved in SUMOylation or another protein with a SUMO-related function. It is also possible that *smt3*/+; *tna*/+ embryos derived from *smt3* mothers do not present the held-out wings phenotype because the SUMOylation pathway can compensate even with low dosages of SUMO. On the other hand, if TnaA is related to SUMOylation, embryos derived from *tna* mothers would lack correct SUMOylation of specific targets (such as Osa) causing later the appearance of the held-out wings phenotype.

Why do *tna* mutant animals die at later stages of development? One possibility is that proteins other than TnaA can exert its function on particular targets, such as Osa, or that they could only impact the TnaA targets in earlier stages of development, but not in later stages. SUMO is required for metamorphosis [29]. As the majority of *tna* mutant animals die as larvae or pupae and cannot proceed to metamorphosis (Fig. 3C) [5], and as TnaA is in prothoracic gland nuclei of third instar larvae (Fig. 2D) obvious candidates for regulation by *tna* would be the ecdysone-pathway, ecdysone-regulated or patterning genes.

The relevance of SUMOylation (and of genes like *tna*) in different developmental processes is just starting to emerge. The requirement of SUMOylation and of *tna* to maintain gene expression makes that the next challenges will be to find the SUMOylation and *tna* targets *in vivo* and to understand the consequences of this modification in proteins involved in chromatin dynamics and in gene expression.

## Supporting Information

Figure S1 **The TnaA_XSPRING_ and Osa antibodies immunoprecipitate TnaA and Osa proteins, respectively. (A)** TnaA was immunoprecipitated from 3–21 hour embryo-soluble nuclear fraction (500 µg) using TnaA_XSPRING_ antibody (1 µg). The Western was revealed with TnaA_XSPRING_ (1∶100). The three panels correspond to films with increasing exposure times. Input (In), Preclearing 1 (Pcl1), Unbound (Ub), and Bound (B). Mock (M) where the immunoprecipitation was done with the equivalent amount of a preimmune serum instead of TnaA_XSPRING_. **(B)** Osa protein was immunoprecipitated from 3–21 hour embryos soluble extract (3.7 mg) with the Osa antibody (1 µg). For Osa detection, the Western was revealed with Osa antibody (1∶1000). Lanes are labeled as above. The equivalent amount of an irrelevant antibody was used as mock. Molecular weight markers are indicated (left).
(TIF)

Figure S2 **Negative controls of TnaA and Osa immunoprecipitations. (A)** TnaA antibodies do not coimmunoprecipitate Hsp70 (Bound, lane B) from a 3–21 hour embryos soluble nuclear fraction (immunoprecipitation shown in Fig. S1A), meanwhile Hsp70 is present in the input (In) and unbound (Ub) samples. The other lanes are preclearing 1 (Pcl1), and mock (M) samples. **(B)** The Osa antibody do not coimmunoprecipitate Cdk7 (immunoprecipitation shown in Fig. S1B). The assays were done as in (A). Lanes are labeled as above.
(TIF)
