# Supplementary figures and images for: TnaA, an SP-RING Protein, Interacts with Osa, a Subunit of the Chromatin Remodeling Complex BRAHMA and with the SUMOylation Pathway in Drosophila melanogaster
Source: PLoS One. 2013 Apr 19;8(4):e62251. doi: 10.1371/journal.pone.0062251 (PMC3631182; doi:10.1371/journal.pone.0062251)

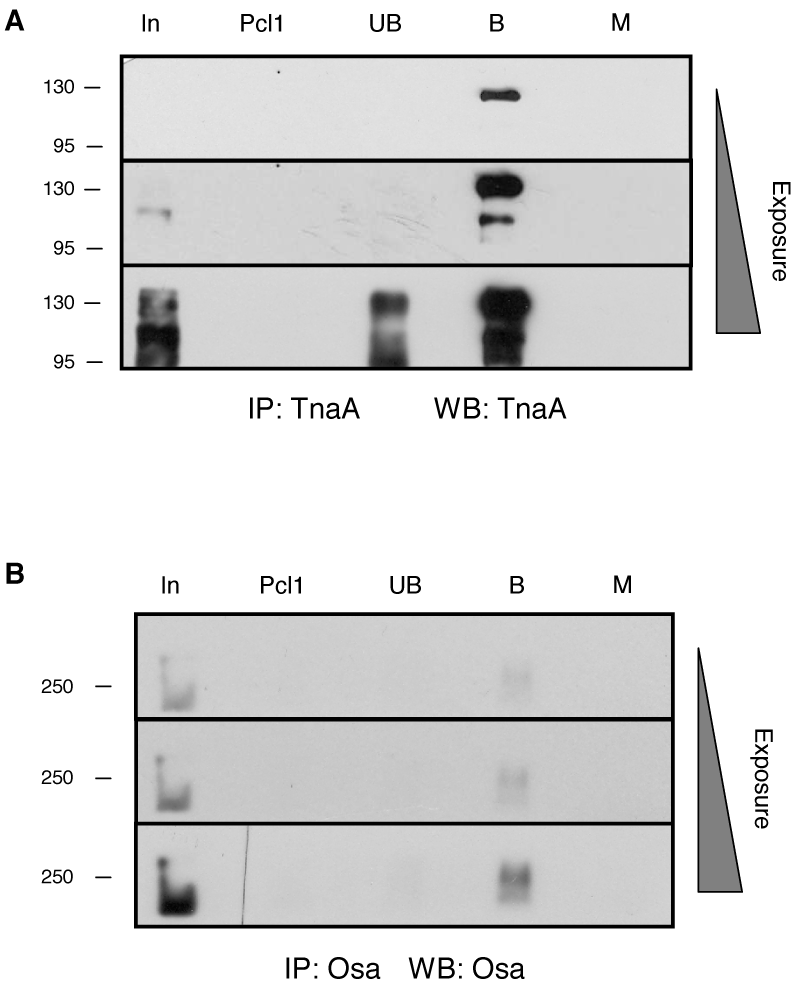

Supplement: Figure S1 — The TnaAXSPRING and Osa antibodies immunoprecipitate TnaA and Osa proteins, respectively. (A) TnaA was immunoprecipitated from 3–21 hour embryo-soluble nuclear fraction (500 µg) using TnaAXSPRING antibody (1 µg). The Western was revealed with TnaAXSPRING (1∶100). The three panels correspond to films with increasing exposure times. Input (In), Preclearing 1 (Pcl1), Unbound (Ub), and Bound (B). Mock (M) where the immunoprecipitation was done with the equivalent amount of a preimmune serum instead of TnaAXSPRING. (B) Osa protein was immunoprecipitated from 3–21 hour embryos soluble extract (3.7 mg) with the Osa antibody (1 µg). For Osa detection, the Western was revealed with Osa antibody (1∶1000). Lanes are labeled as above. The equivalent amount of an irrelevant antibody was used as mock. Molecular weight markers are indicated (left). (TIF) [file pone.0062251.s001.tif]

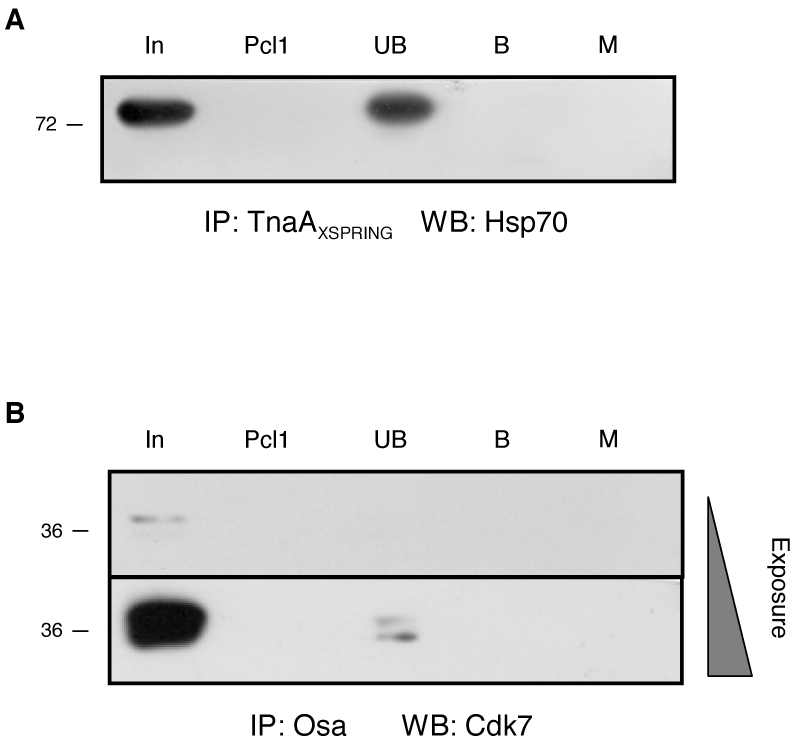

Supplement: Figure S2 — Negative controls of TnaA and Osa immunoprecipitations. (A) TnaA antibodies do not coimmunoprecipitate Hsp70 (Bound, lane B) from a 3–21 hour embryos soluble nuclear fraction (immunoprecipitation shown in Fig. S1A), meanwhile Hsp70 is present in the input (In) and unbound (Ub) samples. The other lanes are preclearing 1 (Pcl1), and mock (M) samples. (B) The Osa antibody do not coimmunoprecipitate Cdk7 (immunoprecipitation shown in Fig. S1B). The assays were done as in (A). Lanes are labeled as above. (TIF) [file pone.0062251.s002.tif]
